# Development of risk prediction model for cognitive impairment in patients with coronary heart disease: A study protocol for a prospective, cross-sectional analysis

**DOI:** 10.3389/fcvm.2022.1107544

**Published:** 2023-01-16

**Authors:** Qing Wang, Shihan Xu, Fenglan Liu, Yanfei Liu, Yue Liu, Fengqin Xu

**Affiliations:** ^1^The Second Department of Geriatrics, Xiyuan Hospital, China Academy of Chinese Medical Sciences, Beijing, China; ^2^National Clinical Research Center for TCM Cardiology, Xiyuan Hospital, China Academy of Chinese Medical Sciences, Beijing, China; ^3^School of Clinical Medicine, Guangdong Pharmaceutical University, Guangzhou, China

**Keywords:** coronary heart disease, cognitive impairment, risk factors, prediction model, cross-sectional analysis

## Abstract

**Background:**

Ischemic heart disease and degenerative encephalopathy are two main sources of disease burden for the global elderly population. Coronary heart disease (CHD) and cognitive impairment, as representative diseases, are prevalent and serious illnesses in the elderly. According to recent research, patients with CHD are more likely to experience cognitive impairment and their cognitive ability declines more quickly. Vascular risk factors have been associated with differences in cognitive performance in epidemiological studies, but evidence in patients with CHD is more limited. Inextricably linked between the heart and the brain. Considering the unique characteristics of recurrent cognitive impairment in patients with CHD, we will further study the related risk factors. We tried to investigate the potential predictors of cognitive impairment in patients with CHD through a prospective, cross-sectional study.

**Methods:**

The cross-sectional study design will recruit 378 patients with CHD (≥65 years) from Xiyuan Hospital of China Academy of Chinese Medical Sciences. The subjects' cognitive function is evaluated with MoCA scale, and they are divided into cognitive impairment group and normal cognitive function group according to the score results. Demographic data, disease characteristics (results of coronary CT/ angiography, number of stents implanted, status of diseased vessels), laboratory tests (biochemistry, coagulation, serum iron levels, pulse wave velocity), metabolites (blood samples and intestinal metabolites), and lifestyle (smoking, alcohol consumption, sleep, physical activity) will be assessed as outcome indicators. Compare the two groups and the correlation analysis will be performed on the development of mild cognitive impairment. Mann-Whitney U or X^2^ test was selected to describe and evaluate the variation, and logistics regression analysis was employed to fit the prediction model. After that, do the calibration curve and decision curve to evaluate the model. The prediction model will be validated by a validation set.

**Discussion:**

To explore the risk factors related to mild cognitive impairment (MCI) in patients with CHD, a new predictive model is established, which can achieve advanced intervention in the occurrence of MCI after CHD. Owing to its cross-sectional study design, the study has some limitations, but it will be further studied by increasing the observation period, adding follow-up data collection or prospective cohort study. The study has been registered with the China Clinical Trials Registry (ChiCTR2200063255) to conduct clinical trials.

## 1. Introduction

With the deepening of population aging, the incidence of cardiovascular and cerebrovascular diseases is increasing annually, which brings heavy social and economic burden. Coronary heart disease (CHD) and cognitive impairment are major diseases that endanger human health, especially for the elderly. Cognitive impairment includes varying degrees from mild cognitive impairment (MCI) to dementia. When memory or other cognitive functions are impaired but not to the point of dementia, this is referred to as MCI (1). Epidemiological investigation have shown that 10–15% of MCI patients develop dementia each year among people over 65 years old (2), while about 70 % of Alzheimer's disease (AD) patients in China are evolved from MCI (3). Hence, it has significant clinical value for the early recognition, early diagnosis and transformation prediction of the disease.

Studies have shown that those with CHD are more likely to experience cognitive impairment. Compared to the general population, the prevalence rate is much greater at roughly 35–46% (4–6). A clinical study published in The New England Journal found that the incidence of cognitive decline in patients with CHD was 42% five years after coronary-artery bypass grafting (CABG) (6). A significant longitudinal cohort research with 7,888 participants (mean age 62.1 ± 10.2 years) indicated that CHD incidents were linked to accelerated cognitive decline. The decline in cognitive ability (including overall cognitive, verbal memory, and time orientation scores) occurred in the years after the diagnosis CHD (7). Similar findings were made by other researchers who discovered that after a follow-up of 3.2 ± 0.37 years, patients with stable CHD had a 42.4% incidence of cognitive impairment (4). In view of this, our study intends to focus on elderly patients with a history of coronary heart disease over 3 years, because of the high incidence of MCI at this stage. Additionally, cardiovascular disease risk factors such as hyperlipidemia, hypertension, diabetes, obesity, smoking and so on are also risk factors of cognitive impairment (8, 9). Clinical observation revealed that cognitive impairment was more pronounced in patients with significant three-vessel disease or coronary artery stenosis (10). Researchers discovered that people with a history of myocardial infarction had a 2–5 times higher chance of developing dementia (7).

The impact of cardiovascular event chain (from cardiovascular risk factors to end-stage heart diseases) characterized by coronary atherosclerosis has expanded to organs outside the heart, particularly the brain, and affects cognitive performance (11, 12). It is of great significance to further study the relationship between CHD and cognitive impairment. Therefore, our study soughts to assess the correlation between CHD and MCI in the elderly by examining the differences of coronary heart disease and its risk factors between patients with cognitive impairment and patients with normal cognition. At the same time, a risk prediction model will be established to provide reference for early clinical identification and intervention of cognitive impairment in patients with CHD. In order to achieve the objective of preventing risk factors from occurring and enhancing cognitive performance.

## 2. Methods and analysis

### 2.1. Study design and participants

**Figure 1:**
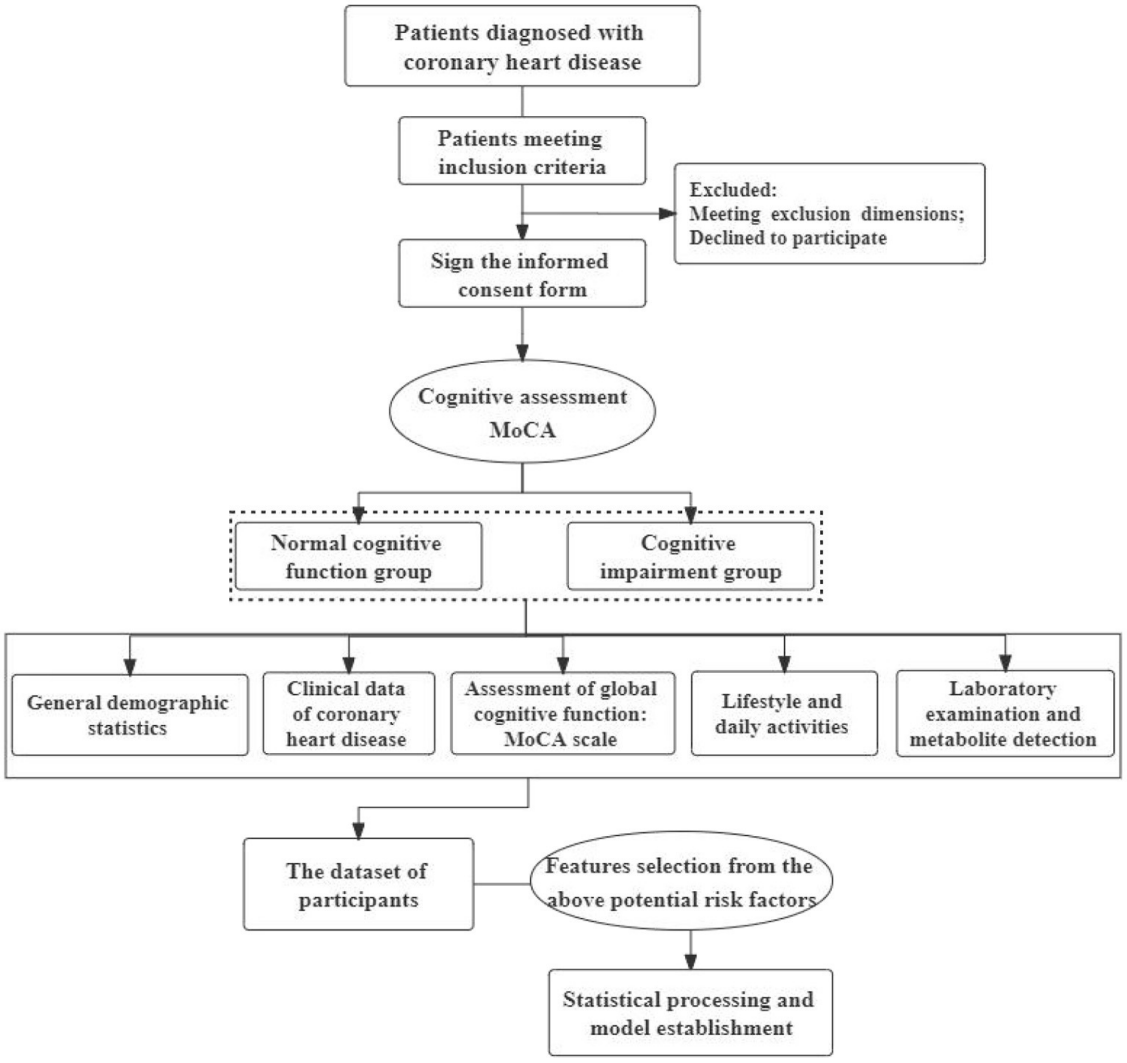
Flow chart of specific steps.

This is a prospective, cross-sectional study with 378 participants. After screening according to the inclusion and exclusion criteria, the informed consent of the subjects was obtained. According to whether CHD patients are accompanied by mild cognitive impairment, the researchers will divide them into a cognitive impairment group (observation group) and a normal cognitive function group (control group). It is based on the program “Research on the Transformation and Application of TCM in the Prevention and Treatment of Secondary Cognitive Impairment of Coronary Heart Disease” sponsored by the Chinese Academy of Chinese Medical Sciences (CI2021A01406) to identify the risk factors for cognitive impairment in CHD patients. The fieldwork for this study will be conducted at Xiyuan Hospital of China Academy of Chinese Medical Sciences in China. Both outpatients and inpatients are allowed to be included. Depending on the feasibility and specific implementation, additional hospital sites may be added. In addition, this study has been registered as a clinical trial by the Chinese Clinical Trials Registry (ChiCTR2200063255), which further standardizes our research. The specific overview of study procedure is shown in Figure 1.

### 2.2. Recruitment and screening

Eligible patients were identified according to the diagnostic description and the International Classification of Diseases (10th and 11th editions). The recruitment of subjects was carried out through offline and online announcements, focusing on offline treatment process. Eligible patients are expected to be enrolled between September 2022 and December 2023. The diagnostic criteria of CHD refer to “guidelines for diagnosis and treatment of stable coronary heart disease” (13) and “guidelines for emergency rapid diagnosis and treatment of acute coronary syndrome (2019)” (14).

For the diagnostic criteria of MCI, refer to the Chinese guidelines on mild cognitive impairment (15). Specifically: (i) cognitive impairment was reported by patients or insiders, or experienced clinicians found cognitive impairment; (ii) objective evidence of impairment of one or more cognitive domains (from cognitive tests); (iii) sophisticated instrumental daily abilities might be slightly hampered, but still preserve independent daily living abilities; iv unable to reach the diagnosis of dementia. Auxiliary diagnostic criteria: the score of Montreal Cognitive Assessment (MoCA) scale <26 points; the score of Clinical Dementia Rating (CDR) scale was 0 or 0.5. MoCA: the full score of the scale is 30, and the score ≥26 is normal. If the subjects' years of education are <12 years (senior high school level), the results can be increased by one point.

All the above are diagnosed by the attending physician. Under the premise of full informed consent, it will be screened by the researcher or the attending physicians according to the requirements. The inclusion and exclusion criteria for this study are shown in Table 1.

**Table 1:** Inclusion criteria and exclusion criteria of the study.

**Inclusion criteria**
Age ≥ 65 years
Meet the diagnostic criteria of CHD, and the medical history ≥3 years
Visual and auditory discrimination is relatively intact, enough to cooperate with relevant tests
Voluntarily participate in the study and sign informed consent form
**Exclusion criteria**
Alzheimer's disease or other types of dementia
Patients with neurological and mental disorders, such as severe depression, schizophrenia, bipolar disorder, etc.
Have suffered from other diseases that affect cognitive function, such as cerebrovascular history, hypothyroidism, obvious folic acid and vitamin B12 deficiency, or previous intracranial surgery
Patients with severe heart disease and perioperative hemodynamic instability other than CHD, primary diseases with important organs, blood system or endocrine system
History of alcohol dependence and special drug abuse, including drugs^*^ that can cause cognitive changes
Patients with severe hepatic and renal insufficiency
Had been diagnosed with cognitive impairment or dementia before the first diagnosis of CHD
Participating in other clinical trials

* Drugs affecting cognitive function mainly include the following categories: anticholinergics, antiepileptics, antiparkinson's, antihistamines, tricyclic antidepressants, skeletal muscle relaxants.

### 2.3. Sampling and sample size

For the exploratory analysis of multiple risk factors, the sample size of this study is set to be >5–10 times the number of observed factors (16, 17). According to previous research reports, after 3.2 ± 0.37 years of follow-up, the incidence of cognitive impairment in patients with stable coronary heart disease is 42.4% (4). Clinical observations showed that patients with CHD had a relatively higher incidence of MCI. Based on the actual conditions of patients in the hospital, and the 18 independent variables pre-estimated in the study (detailed in the Outcomes Measures part), the sample range should be at least 90–180. Considering the absence and effectiveness of 5% of the samples, the total sample size finally determined by the two groups should ≥378. The final sample size of each group shall be determined according to the actual situation and can be adjusted appropriately.

### 2.4. Outcomes measures

Clinical, biological, social and environmental determinants of interest in this study were selected based on the potential or established association with cognitive impairment in patients with CHD and/or the fact that they are modifiable through preventive strategies.

#### 2.4.1. Demographic and clinical data

Standardized questionnaires will be used to collect demographic, symptom, and medical history data. Social demographic information includes age, gender, race, height, weight, BMI, marital status, education level, nature of work, and type of occupation. Clinical data include past and present diseases (acute and chronic), drugs, and family history of cardiovascular disease. Disease-related information includes the course of CHD, the results of coronary CT/ angiography, the number of coronary stents implanted, cardiac function class, the number of diseased vessels, types of statins, and drug treatment. Previous disease history and drug use also need to be recorded. Vital signs such as temperature, respiration, pulse and blood pressure are also covered.

#### 2.4.2. Cognitive assessment

At baseline, all participants underwent cognitive assessment. Global cognitive function will be assessed using the MoCA scale. Neuropsychological scale screening tests are crucial in the identification of cognitive impairment. Currently, the Mini-Mental State Examination (MMSE) and Montreal Cognitive Assessment (MoCA) are the most commonly used cognitive assessment tools. Of the two, MoCA covers a wider variety of cognitive domains than MMSE. In addition, meta-analysis findings (18) revealed that the sensitivity, specificity and AUC value of MoCA are higher than those of MMSE in elderly patients (over 60 years old) with MCI. Therefore, we choose MoCA as the screening method to determine whether there is MCI.

As a screening tool specially developed to detect MCI, it takes around 10 min to administer MoCA when executed (19). The MoCA covers 14 topics with a total of 30 points, including visual spatial structure skills, executive function, attention and concentration, short-term memory and delayed recall, language, abstract thinking, calculation and orientation. Due to varied geographies, cultures and populations, there are some differences in the cut-off values of MoCA screening for MCI among elderly individuals in various regions. Studies have shown that when using a cutpoint of 25/26, the MoCA has a sensitivity of 80–100% and specificity of 50 to 76% for detecting MCI (20). According to a survey to evaluate MCI in Beijing, China's urban and rural old communities, MoCA has excellent sensitivity and fairness specificity under the recommended critical score of 26 (21). The systematic review of MCI screening for the elderly in China also concurs that using a diagnostic threshold of 25/26 is the most practical option (22). Thus, the score of MoCA <26 is chosen as the benchmark to ascertain whether MCI exists in this study.

#### 2.4.3. Health and lifestyle

According to the investigation, researchers found that an insomnia history was associated with an increased risk of MCI, and a sleep disorder history may usefully predict MCI (23). Therefore, we specially selected the commonly used Pittsburgh Sleep Quality Index (PSQI) to analyze the sleep characteristics of the subjects. Sleep quality, falling asleep time, sleep time, sleep efficiency, sleep disorders, hypnotic drugs and daytime dysfunction are included in this scale. The World Health Organization (WHO) guidelines recommend that elderly people exercise at least 150 min medium intensity or 75 min high intensity aerobic exercise per week. So the physical activity of the participants will also be monitored to explore if it is related to MCI. Physical activity levels will be measured by using a Chinese version of the International Physical Activity Questionnaire-short form (IPAQ-S) (24).

#### 2.4.4. Laboratory indicators

Laboratory examination can accept the test results within 7 days before and after screening. It mainly includes the following contents: fasting glucose, glycosylated hemoglobin (HbA1c), total cholesterol, triglyceride, low-density lipoprotein, high-density lipoprotein, total bilirubin, serum creatinine, serum iron level (Fe), coagulation function (PT, APTT, TT, FIB), vascular ultrasound pulse wave velocity (PWV).

In addition to the above-mentioned indexes, in the early morning of the 2nd day after the selection, two tubes of peripheral venous blood of 5 ml will be drawn from a qualified phlebotomist and placed in the EDTA anticoagulant tubes. Centrifuge the blood samples (3,000 rpm at 4°C for 15 min), store them at −80°C, and then conduct untargeted metabolomics detection. 2 g of fresh fecal samples will be collected and put into aseptic collection tube and stored at −80°C immediately for metabolic detection and analysis of intestinal flora.

### 2.5. Quality control

All researchers can enter and conduct this trial only after they have completed the training and obtained the authorization of the principal researcher. The involved researchers were trained in Good Clinical Practice (GCP) prior to the start of this study. Every researcher has received training in research procedure and plan. Including the collection of the MoCA scale, the researchers mastered the relevant questioning skills and evaluation criteria after strict and standardized training in advance. Ensure the rigor and consistency of the implementation process. All investigators have a thorough understanding of the information in this clinical research protocol, master the GCP principles, unify the recording methods and judgment standards, and strictly follow the protocol.

### 2.6. Statistical analysis

Among the outcomes of this study, the measurement data in accordance with normal distribution are expressed by mean ± standard deviation, and independent sample *t*-test is used for comparison between the two groups. The measurement data that don't conform to the normal distribution are expressed in the median (quartile), and the Mann-Whitney U test is used for comparison between the two groups. Counting data were expressed as frequency or percentage, and X^2^ test was used to compare the two groups. Single factor analysis shall be conducted for all factors. In order to avoid the omission of important variables, the covariates with P <0.1 in the univariate analysis will be included in the multivariate logistic regression analysis. Stepwise forward regression analysis is used to analyze the independent risk factors of cognitive impairment in patients with CHD, and the OR value and 95% CI of each related factor will be estimated. The risk prediction model of cognitive impairment in patients with CHD would be established by adding independent risk factors. ROC curve will be drawn and the area under the curve (AUC) will be calculated to evaluate the effectiveness of the prediction model. Use Bootstrapping method to verify the accuracy of the final model internally. All tests with P<0.05 on both sides indicate that the difference is statistically significant, and other statistical analysis can be carried out by SPSS25.0.

### 2.7. Data management

In this study, standardized case report form (CRF) tables will be used to collect data. Each participant will be assigned a unique identifier with the hospitalization number or patient ID code. Medical and other records of the participants will be kept by the researchers. EpiData3.1 software will be used for data entry and establishing the database. Once the data input is completed, the integrity and reasonableness of all data will be evaluated and checked by the researchers item by item. Check the problem with the original survey data and correct it after confirmation.

## 3. Discussion

Age-related cognitive decline is a series of natural cognitive changes that may develop into MCI or dementia. MCI is a state of cognitive impairment between healthy aging and dementia, which is considered as the prophase of dementia (25). But at this stage, the function of daily life remains basically intact. Delaying the onset of cognitive decline is the optimal strategy but where there are some earlier measures to slow the decline is important. Through comparative analysis of the population characteristics of the two groups, our study will screen out the specific markers of cognitive impairment in patients with CHD. So as to provide reference for early clinical identification and intervention of the disease.

The objective of this study is to ascertain the relationship between CHD and its risk factors and cognitive impairment in the elderly. At the same time, establish the risk prediction model of cognitive impairment in patients with CHD. The study will make available data regards CHD and MCI risk prediction. We propose leveraging the progress of machine learning to systematically compare different modeling approaches (26) to develop predictive tools for cognitive impairment in patients with CHD and verify their effectiveness. Comprehensive application of clinical observation, multi-source data processing, machine learning and other methods is also the innovation of the study.

Still, this research is subject to certain limitations. As an observational study, cross-sectional design has limitations e.g., causality cannot be inferred, we can only address the association. However, we try to include more observation factors, which are widely related to basic demographic data, disease characteristics, blood chemistry, metabolites and lifestyle. It is expected that some risk factors with clinical value can be found on the basis of this exploratory analysis. In order to broaden the current knowledge on CHD and its relationship with cognitive impairment in the elderly. Additionally, it will serve as a valuable foundation for hypothesis generation for future longitudinal studies and / or randomized controlled trials. In the future, it is necessary to conduct a larger sample of prospective cohort studies and add follow-up time to determine the incidence of secondary MCI in patients with CHD.

## Ethics statement

The study was approved by the Ethics Committee of Xiyuan Hospital of Chinese Academy of Chinese Medical Sciences (2022XLA060-3). This study involves elderly patients with cognitive impairment and the legitimate rights and interests of the subjects should be fully protected. Trained researchers will provide the subjects with informed consent forms, which are easy to understand and clear. Researchers will patiently and completely explain the purpose, procedure, potential benefits, and risks of this study before each subject is recruited for it. The principles of complete information disclosure and voluntary choice must be adhered to during the informed consent procedure. Subjects have the right to refuse to participate without giving reasons. After participants have voluntarily signed the informed consent form, attention will be paid to the questioning skills during the study, so as to avoid the unfamiliar emotions of the elderly subjects due to their participation in clinical research. Any modification of the research protocol will be submitted to the Research Ethics Committee for approval. Currently, the researchers are recruiting participants. The research results of this study will be published in medical journals in Chinese or English, but the patients' information will be kept private as required by law. When necessary, government administrative departments, hospital Ethics Committees, and their relevant personnel may consult the patient's data according to the regulations.

## Author contributions

FX and YuL conceived and designed the study protocol, helped to critically revise the draft, and conduct the final manuscript. QW, SX, and FL drafted the manuscript. YaL and QW made critical revision. All authors contributed to this article and approved the submitted version.
